# Anatomy Gone Wrong: A Rare Tale of Superior Mesenteric Artery Syndrome and Duodenal Obstruction

**DOI:** 10.7759/cureus.86345

**Published:** 2025-06-19

**Authors:** Soraya Gioftsiou, Malak Faiz, Mohamed Mohammadi

**Affiliations:** 1 Hepato-gastroenterology, Cheikh Zaid University Hospital, Rabat, MAR; 2 Gastroenterology, Cheikh Zaid Teaching Hospital, Rabat, MAR

**Keywords:** case report, duodenal obstruction, duodenal stenosis, laparoscopic surgery, occlusion, superior mesenteric artery syndrome

## Abstract

Aorto-mesenteric compression syndrome, also known as superior mesenteric artery (SMA) syndrome, is a rare but potentially serious condition caused by compression of the third portion of the duodenum between the abdominal aorta and the SMA. This anatomical narrowing leads to partial or complete duodenal obstruction and is often associated with significant weight loss and gastrointestinal symptoms. We present a case of a 16-year-old male who experienced four months of progressive postprandial vomiting, early satiety, and an 8 kg weight loss. On examination, he appeared mildly undernourished (BMI: 18) with epigastric tenderness. Laboratory tests revealed hypokalemia and metabolic acidosis. Abdominal contrast-enhanced CT demonstrated a significantly reduced aorto-mesenteric angle (12°) and compression of the left renal vein with a hilum-to-SMA diameter ratio of 7.4, consistent with the diagnosis of both SMA syndrome and nutcracker syndrome. Conservative treatment with nutritional support and positional therapy failed to alleviate symptoms. The patient subsequently underwent laparoscopic gastrojejunostomy, resulting in clinical improvement and resolution of vomiting. This case highlights the importance of considering SMA syndrome in adolescents presenting with chronic vomiting, weight loss, and nonspecific upper abdominal symptoms. Timely diagnosis through cross-sectional imaging is crucial, and surgical intervention should be pursued when conservative management proves ineffective.

## Introduction

Superior mesenteric artery (SMA) syndrome, also referred to as Wilkie’s syndrome or aorto-mesenteric compression syndrome, is a rare clinical entity characterized by extrinsic compression of the third portion of the duodenum between the aorta and an acutely angulated SMA. This anatomical alteration leads to functional duodenal obstruction and a constellation of nonspecific gastrointestinal symptoms, including early satiety, postprandial abdominal pain, nausea, and bilious vomiting [1,2].

The condition is most commonly seen in adolescents and young adults who experience rapid or severe weight loss due to eating disorders, chronic illness, trauma, or surgery, leading to loss of the retroperitoneal fat pad that cushions the SMA [3,4]. The prevalence is estimated between 0.013% and 0.3%, making it an underrecognized cause of intestinal obstruction [5].

Diagnosis is primarily radiologic, with contrast-enhanced CT or magnetic resonance angiography revealing a reduced aorto-mesenteric angle (normally 25-60°, narrowed to <22° in SMA syndrome) and shortened aorto-mesenteric distance (<8 mm) [6]. This syndrome can be associated with nutcracker syndrome, a compression of the left renal vein by the same vascular structures, potentially leading to hematuria and flank pain [7].

Given its rarity and nonspecific symptoms, SMA syndrome often poses a diagnostic challenge and may be overlooked. Timely recognition is critical to avoid complications such as electrolyte imbalances, malnutrition, and intestinal perforation. Management typically begins with conservative nutritional therapy, although surgical intervention may be required in refractory cases [8].

## Case presentation

Patient and observation

Patient Information

A 16-year-old patient with no significant medical history presented for consultation due to immediate postprandial vomiting of an alimentary nature that had persisted for the past four months. This symptom was accompanied by an unintended weight loss of 8 kg, raising concerns about an underlying pathological condition.

Clinical Findings

Upon clinical examination, the patient had a body mass index (BMI) of 18, suggesting mild undernutrition. Physical examination revealed tenderness in the epigastric region, which indicated possible gastrointestinal involvement. Additionally, a left-sided varicocele was noted; however, there was no evidence of hematuria. Laboratory tests conducted upon admission showed hypokalemia associated with hypochloremic acidosis, which was promptly managed through appropriate hydration and potassium supplementation.

Diagnostic Assessment

**Figure 1:**
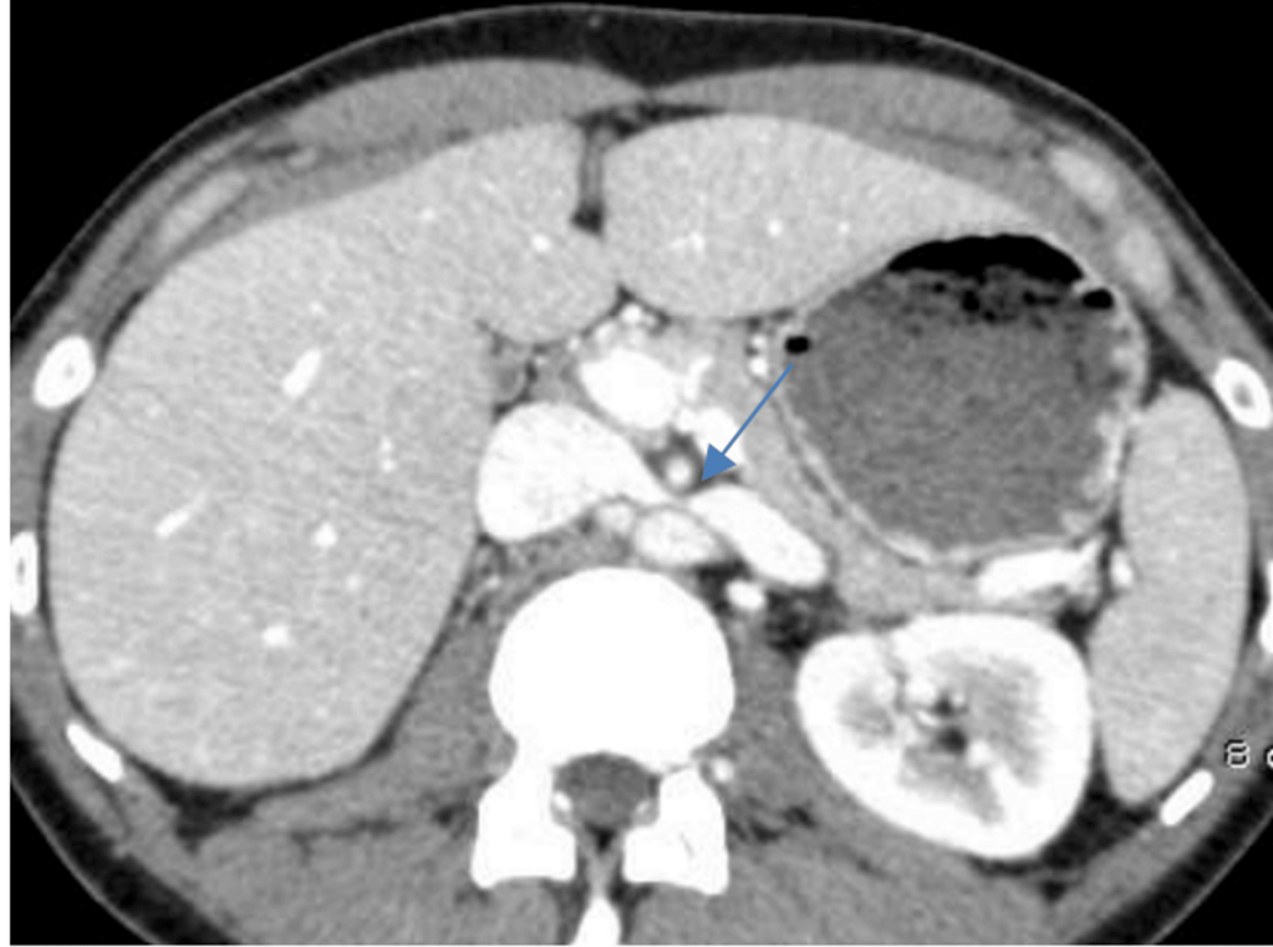
Axial section of an abdominal-pelvic CT scan after contrast injection. Axial section of an abdominal-pelvic CT scan after contrast injection showing a narrowing of the left renal vein in the aorto-mesenteric fork, forming the "beak sign" (arrow), with the ratio of the hilum portion diameter to the aorto-mesenteric portion of the left renal vein measuring 7.4 (>4.9).

Given the persistence and nature of the symptoms, an abdominal-pelvic CT scan was performed to identify any anatomical or vascular abnormalities. The imaging revealed dilation of the left renal vein, with a ratio of the diameter of the hilum portion to the aorto-mesenteric portion measuring 7.4, which was significantly above the threshold of 4.9 (Figure 1).

**Figure 2:**
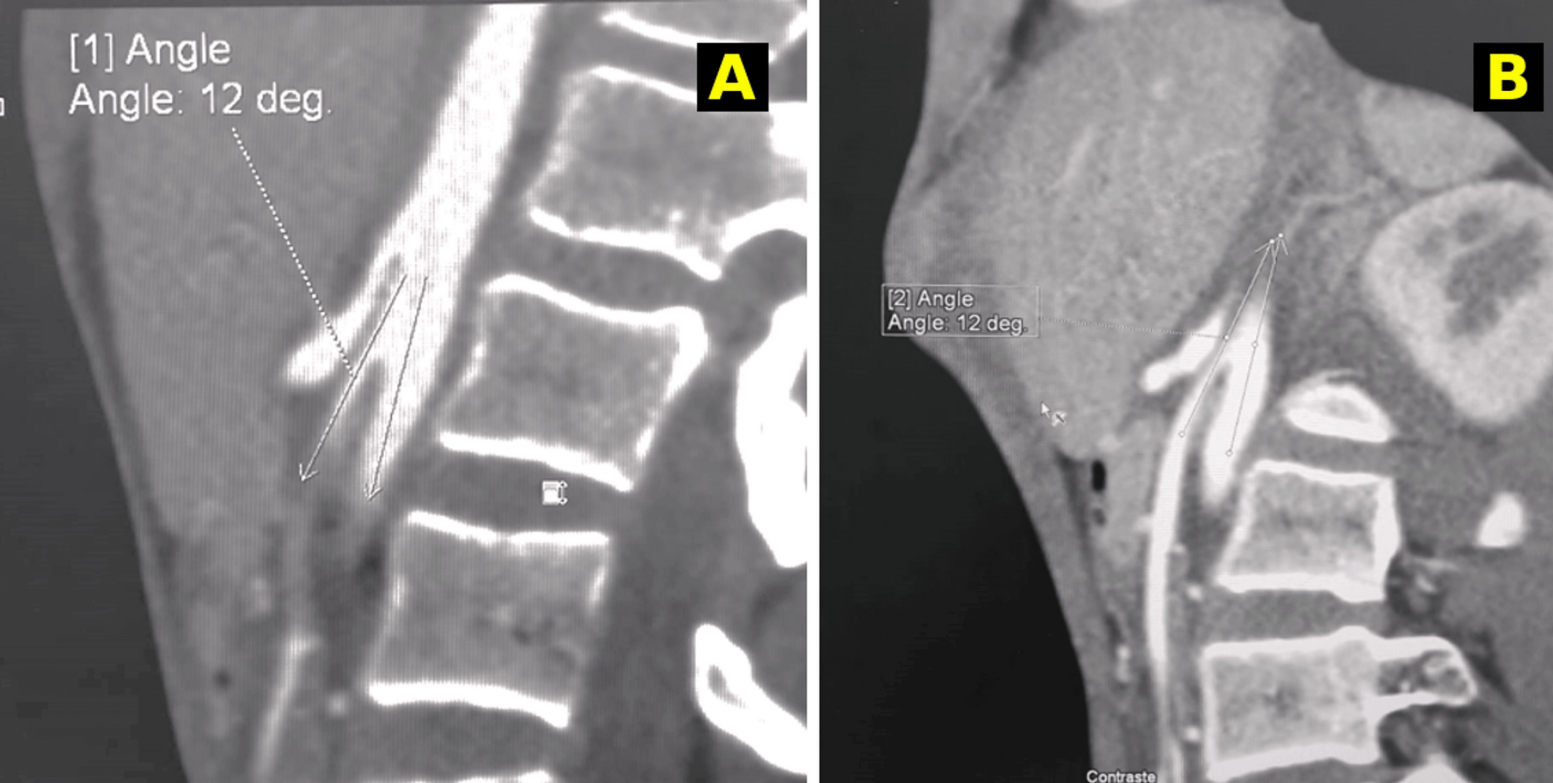
Sagittal section of an abdominal-pelvic CT scan after contrast injection. Sagittal reconstruction from a contrast-enhanced abdominal CT scan demonstrating a markedly reduced aorto-mesenteric angle, measured at 12°, consistent with superior mesenteric artery (SMA) syndrome. (A) Sagittal CT slice showing the compression of the third portion of the duodenum between the abdominal aorta and the SMA, with measurement lines indicating the narrowed angle. (B) Another sagittal view confirming the aorto-mesenteric angle reduction to 12°, supporting the diagnosis of SMA syndrome.

Furthermore, there was a marked narrowing of the left renal vein as it became compressed within the fork formed by the abdominal aorta and the SMA. The angle between these two arteries was measured at 12°, which is notably lower than the normal value of 41° (Figure 2).

These findings were suggestive of nutcracker syndrome, a vascular compression disorder.

Therapeutic Intervention

Initial management involved conservative medical treatment, including rehydration and correction of electrolyte imbalances. Despite four days of symptomatic treatment, the patient showed no significant improvement in symptoms, necessitating a surgical approach. A laparoscopic gastrojejunostomy was performed successfully, and the postoperative course was uneventful. The patient tolerated the procedure well, and there were no immediate complications.

Follow-Up and Outcomes

Following surgery, the patient's clinical symptoms improved notably. The blood work also normalized, with a follow-up potassium level of 4.2 mmol/L and normal renal function. By the second postoperative day, bowel transit had recovered, and a gradual reintroduction of oral feeding was initiated. The patient tolerated oral intake well, demonstrating a positive response to the intervention. Given the favorable recovery, the patient was discharged from the hospital after seven days in stable condition. At the 15-day and one-month follow-up visits, the patient maintained excellent tolerance to oral feeding, with no reported nausea or vomiting, and a weight gain of 4 kg was observed.

## Discussion

The aorto-mesenteric pinch syndrome (PAM) was first described by Rokitansky in 1861, followed by a series of 75 cases published by Wilkie in 1921 [9]. This rare condition results from the compression of the duodenum by the SMA, often related to a reduction of the aorto-mesenteric space to less than 8 mm at the level of the third portion of the duodenum and an aorto-mesenteric angle of less than 20° [1,3,6]. Under normal conditions, this space is kept open by the adipose tissue surrounding the SMA, preventing duodenal compression [2,4,5].

Several etiologies have been identified in the literature, including rapid growth or excessive weight loss, leading to a reduction in the perivisceral fat surrounding the SMA. Other factors, such as a low insertion of the SMA on the aorta or a short Treitz ligament, also contribute to this pathology by modifying the dynamics of the duodenojejunal angle. In our case, the suggested cause was the presence of a short Treitz ligament. Familial forms have also been observed, which may suggest a genetic involvement in some cases [2-4].

Clinically, the syndrome can manifest through various symptoms. A characteristic sign is high intestinal obstruction, which is sometimes atypical, especially in the absence of cessation of stool and gas passage. This clinical presentation may lead to numerous differential diagnoses. Abdominal CT with contrast injection is used to confirm the diagnosis by visualizing the abnormal anatomy. Normally, the distance between the aorta and the SMA ranges from 10 to 28 mm, and the aorto-mesenteric angle is between 45° and 60°. In PAM, CT shows duodenal dilation, a distance of less than 8 mm between the aorta and the SMA, and a reduced aorto-mesenteric angle ranging from 6° to 15° [2,4,6,9].

The prognosis of the syndrome depends on the promptness of the diagnosis and the therapeutic approach. Acute forms can lead to severe complications, such as hydro-electrolytic disturbances, severe malnutrition, and even digestive perforation due to acute duodenal dilation. In our case, the patient presented with a chronic form with significant weight loss and severe hydro-electrolytic disorders [7,9,10].

The initial management is primarily medical, including the correction of ionic imbalances, nutritional support through parenteral or enteral feeding, and the placement of a nasogastric tube for gentle aspiration. Surgery is indicated in cases of failure of well-conducted medical treatment. However, in more than 75% of cases, surgical intervention is necessary. In our patient, due to the chronic evolution, severe hydro-electrolytic disorders, and insufficient response to symptomatic treatment, surgical intervention was decided. Among the different surgical techniques, duodenojejunostomy has shown the best results, which was performed in our case [3,8-11].

In recent years, the integration of the Internet of Things (IoT) into surgical practice has opened new possibilities for improving postoperative monitoring and outcomes. IoT-based systems allow for real-time data collection and patient tracking, which can enhance decision-making and early detection of complications [12].

The outcome is generally favorable, as evidenced by our experience. The patient experienced immediate cessation of vomiting, reduction in pain, and, in the long term, an 8 kg weight gain within three months. The total hospitalization duration was 12 days, which corresponds to the average reported in the literature, with an average hospitalization time of 10 days [6,7,9,11].

## Conclusions

Aorto-mesenteric compression syndrome, though recognized for decades, remains a rare and underdiagnosed cause of upper digestive tract obstruction, particularly in young patients presenting with postprandial vomiting and significant weight loss. While several anatomical variants exist, acquired forms linked to rapid weight loss are most commonly encountered in clinical practice. Diagnosis relies heavily on imaging, especially contrast-enhanced abdominal CT, which allows precise assessment of the aorto-mesenteric angle and duodenal compression. Although conservative management involving nutritional support and postural therapy is the first-line approach, it often fails, making a surgical intervention (most notably duodenojejunostomy) the only treatment providing effective and durable relief, significantly improving quality of life. Clinicians should maintain a high index of suspicion for this condition in appropriate clinical contexts to ensure timely diagnosis and treatment.
